# Maternal smoking and autism spectrum disorder: meta-analysis with population smoking metrics as moderators

**DOI:** 10.1038/s41598-017-04413-1

**Published:** 2017-06-28

**Authors:** Yonwoo Jung, Angela M. Lee, Sherry A. McKee, Marina R. Picciotto

**Affiliations:** 1 Department of Psychiatry, Yale University School of Medicine, New Haven, CT 06508 USA; 2 Interdepartmental Neuroscience Program, Yale University School of Medicine, New Haven, CT 06508 USA

## Abstract

While exposure to nicotine during developmental periods can significantly affect brain development, studies examining the association between maternal smoking and autism spectrum disorder (ASD) in offspring have produced conflicting findings, and prior meta-analyses have found no significant association. Our meta-analysis used a novel approach of investigating population-level smoking metrics as moderators. The main meta-analysis, with 22 observational studies comprising 795,632 cases and 1,829,256 control participants, used a random-effects model to find no significant association between maternal smoking during pregnancy and ASD in offspring (pooled odds ratio (OR) = 1.16, 95% CI: 0.97–1.40). However, meta-regression analyses with moderators were significant when we matched pooled ORs with adult male smoking prevalence (*z* = 2.55, *p* = 0.01) in each country, using World Health Organization data. Our study shows that using population-level smoking metrics uncovers significant relationships between maternal smoking and ASD risk. Correlational analyses show that male smoking prevalence approximates secondhand smoke exposure. While we cannot exclude the possibility that our findings reflect the role of paternal or postnatal nicotine exposure, as opposed to maternal or *in utero* nicotine exposure, this study underlines the importance of investigating paternal and secondhand smoking in addition to maternal smoking in ASD.

## Introduction

Autism is a neurodevelopmental disorder defined by persistent difficulties in reciprocal social interaction and in verbal and nonverbal communication skills. Restrictive and repetitive behavioral symptoms and stereotyped patterns of interest are characterized to varying degrees^1^. The Centers for Disease Control and Prevention estimate that 1 in 68 children have been diagnosed with autism spectrum disorders (ASDs), accounting for more than 3.5 million disability-adjusted life years in America^2^.

ASDs are considered multifactorial disorders that arise from an interaction between genetics and environmental factors. Several genomics studies have revealed single nucleotide polymorphisms, copy-number variations, and *de novo* mutations that are associated with ASD^3–6^. However, other studies highlight the fact that most genetic risk factors, while able to influence the ASD phenotype, cannot independently produce ASD^7, 8^. Current studies have been exploring environmental factors (ex: viral infection, environmental toxins, gestational diabetes) possibly involved in ASD etiology^9^. Identifying controllable environmental risk factors will be important for preventing ASD.

One such environmental risk factor that has garnered interest is smoking tobacco. A combination of animal studies and human epidemiological studies point to a plausible relationship between maternal smoking and ASD in offspring. Nicotine, among the thousands of ingredients of tobacco smoke, is the chemical with the highest likelihood of adversely affecting brain development^10^. Nicotine’s adverse effects are thought to occur via its actions at nicotinic acetylcholine receptors, which mediate neural structural changes^11, 12^, that in turn can have significant consequences for the offspring in later life. In mice, developmental nicotine exposure *in utero* and up to postnatal day 21 significantly affected neuronal dendritic morphology and behavior in a passive avoidance paradigm^11^. Human studies have shown that socioeconomic status (SES) is significantly associated with ASD^13^, and that women with lower SES have a higher tendency to smoke during pregnancy^14^. Additionally, smoking is associated with adverse birth outcomes, such as fetal growth restriction and low birth weight^15^, which are in turn associated with increased risk of ASD^16^. Smoking during pregnancy has also been associated with neurodevelopmental disorders such as attention deficit hyperactivity disorder, conduct disorder, and antisocial behavior^17^.

Epidemiological studies exploring the relationship of maternal smoking during pregnancy and ASD have produced contradictory results. For example, in Swedish populations, Lee *et al*.^18^ found no evidence of an association between maternal smoking and ASD risk, while Larsson *et al*.^19^ found a positive association (OR = 2.09, 95% CI: 1.08–4.03), and Haglund *et al*.^20^ found a negative association (OR = 0.7, 95% CI: 0.5–1.0). Similarly, positive^21, 22^, negative^23, 24^, and null^25–28^ associations have been observed in United States study populations. Three meta-analyses have also been conducted, each finding no evidence for a significant association between maternal smoking and ASD risk in offspring. Gardener *et al*.^29^ included 5 studies and reported an overall OR of 1.00 (95% CI: 0.75–1.36). Rosen *et al*.^30^ included 15 studies and found a summary OR of 1.02 (95% CI: 0.93–1.12), while Tang *et al*.^31^ analyzed a partially different set of 15 studies and found a summary OR of 1.02 (95% CI: 0.93–1.13). However, these prior meta-analyses failed to account for study-level and population-level factors regarding smoke exposure that may moderate the relationship between maternal smoking and ASD risk. Specifically, the studies do not systematically account for the possible role of indirect, secondhand smoke exposure; most of the observational studies included in these meta-analyses only reported active maternal smoking. This assessment alone may inadequately reflect total *in utero* nicotine exposure.

Here, we analyze population smoking prevalence in our meta-regression to investigate how these measures, as potential indicators of secondhand smoke exposure, might moderate the relationship between maternal smoking and ASD. Additionally, two studies^22, 32^ supporting an association between maternal smoking and ASD risk have been published after the most recent meta-analysis. One of the two studies^32^ is the first to report extractable data regarding maternal smoking and ASD in an Asian population. Previous meta-analyses have only included studies conducted in European or North American populations. Therefore, the current meta-analysis also included a subgroup analysis by study location. This study was conducted to assess the association between maternal smoking and ASD risk comprehensively, and to examine study location and population smoking metrics as important study-level and population-level moderators of this relationship.

## Results

### Literature Search and Selection

**Figure 1:**
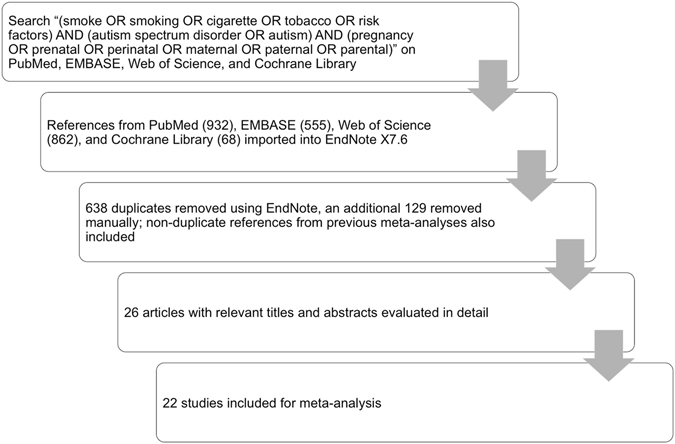
Study selection strategy.

Our search strategy returned a total of 2,417 publications from PubMed, EMBASE, Web of Science, and Cochrane Library databases (Fig. 1). All references were imported into EndNote X7.6 (Thomson Reuters), which automatically removed 638 duplicates. An additional 129 duplicates were removed manually, leaving 1650 articles that were screened by titles and abstracts for relevance to maternal smoking and ASD. Studies included in previously published meta-analyses^30, 31^ were also evaluated for inclusion criteria. After screening, 26 studies remained for detailed evaluation of the full texts. Four articles^33–36^ were then excluded for lacking sufficient data to fill the contingency table of binary maternal smoking and ASD variables. Consequently, 22 articles^17–28, 32, 37–45^ were included for meta-analysis.

### Study Characteristics

The 22 studies analyzed include seven cohort studies^19, 23, 39, 40, 43–45^ and fifteen case-control studies^17, 18, 20–22, 24–28, 32, 37, 38, 41, 42^, with a total of 795,632 cases and 1,829,256 control participants. Nine studies^17–20, 37, 38, 41, 42, 45^ were from Europe (Denmark, Sweden, Finland, The Netherlands, and Poland), twelve studies^21–28, 39, 40, 43, 44^ were from North America (Canada and U.S.) and one study^32^ was from Asia (China). Information on smoking during pregnancy was collected during a prenatal visit or at birth in 11/22 studies, and ASD diagnosis was ascertained by medical records in 16/22 studies (Supplementary Table S1).

### Quality Assessment and Publication Bias

**Figure 2:**
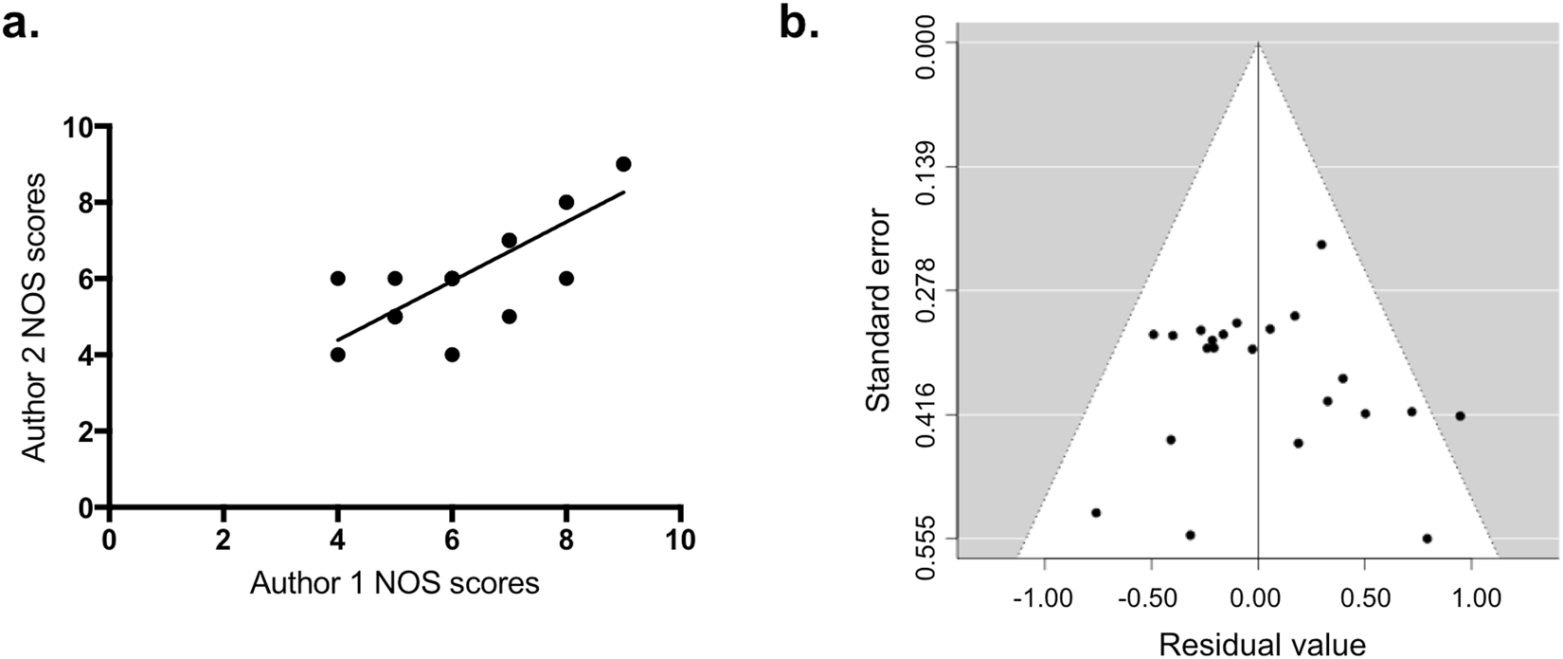
Distribution of studies used in the meta-analysis by (**a**) study quality, as assessed on the NOS by two independent raters with high correlation (*r*(20) = 0.81, *p* < 0.00001), and by (**b**) funnel plot, to diagnose publication bias.

Two authors independently assigned Newcastle-Ottawa Scale (NOS) scores to each study, with high correlation between scorers (*r*(20) = 0.81, *p* < 0.00001) (Fig. 2a). Overall, the studies had moderate methodological quality as scored on a conservatively-interpreted NOS, with an average score of 6.5 and a range of 4–9. Based on thirds of the range of scores, four studies were considered “high” quality with NOS scores of 8 or 9^17, 22, 25, 37^, ten studies were considered “medium” quality with NOS scores of 6 or 7^18, 21, 23, 26, 38–40, 42, 44, 45^, and eight studies were considered “low” quality with NOS scores of 4 to 5.5^19, 20, 24, 27, 28, 32, 41, 43^ (Supplementary Tables S1 and S2).

The shape and symmetry of the funnel plot of the log ORs from the 22 studies are illustrated in Fig. 2b. Begg’s rank correlation test (*p* = 0.27) and Egger’s regression test (*p* = 0.54) suggest no significant publication bias^46, 47^.

### Main Analysis

**Figure 3:**
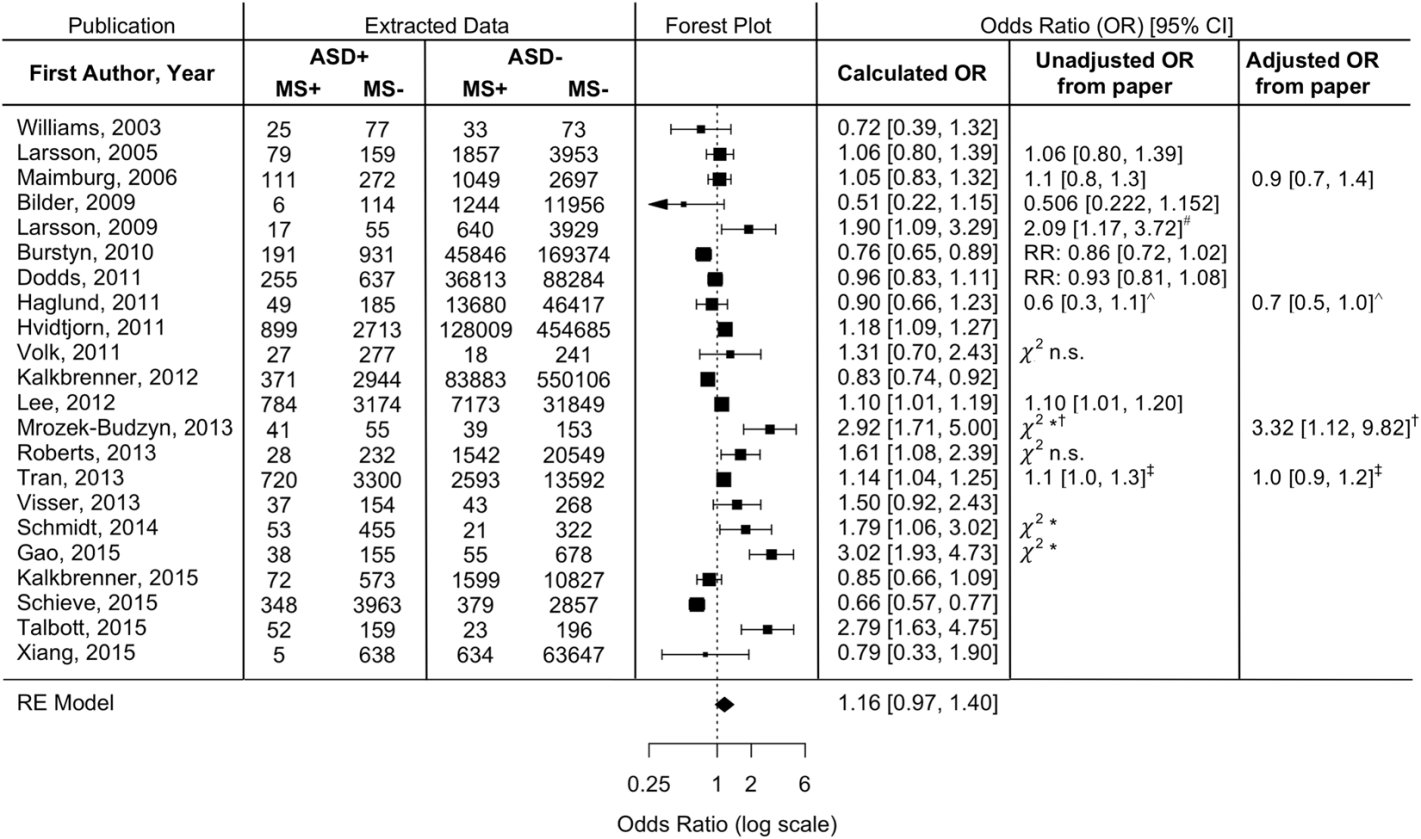
Meta-analysis of the association between maternal smoking during pregnancy and ASD risk based on 22 observational studies. The pooled OR using a random-effects model was found to be 1.16 (95% CI: 0.97–1.40). Data extracted from the papers and ORs calculated from this data are shown, as well as unadjusted and adjusted ORs reported in the observational study publications, or an alternate statistic reported if not ORs. ASD = autism spectrum disorder; MS = maternal smoking during pregnancy; RR = relative risk; *p ≤ 0.05; n.s. = *p* > 0.05. ^#^As reported for “yes” in response to maternal smoking query. Data extracted for meta-analysis combined “yes” and “yes, but stopped” responses. ^∧^As reported for ASD and smoking ≥10 cigarettes/day. Data extracted for meta-analysis combined ASD and Asperger’s subgroups, and smoking 1–9 and smoking ≥10 cigarettes/day. ^†^As reported for active maternal smoking. Data extracted for meta-analysis combined active and passive maternal smoking. ^‡^As reported for smoking in all pregnancy. Data extracted for meta-analysis combined only first trimester and all pregnancy smoking.

The pooled OR estimates showed that maternal smoking during pregnancy was not associated with a significantly increased risk of ASDs (OR = 1.16; 95% CI: 0.97–1.40) (Fig. 3). A statistically significant level of heterogeneity was found across studies (*I* ^2^ = 94.08%, Q(21) = 156.94, p < 0.0001).

### Subgroup and Sensitivity Analyses, and Moderator Analysis with Meta-regression

To identify potential causes of the high heterogeneity in our main analysis, we performed subgroup and sensitivity analyses. Subgroup analyses were stratified by study design, location, sample size, quality, adjustment for confounders, maternal smoking assessment, and ASD status ascertainment (Table 1, Supplementary Tables S1 and S2). Maternal smoking during pregnancy was associated with significantly elevated risk of ASD in European populations (OR = 1.14, 95% CI: 1.08–1.19) and in the single study conducted in Asia (OR = 3.02, 95% CI: 1.93–4.73), but not in North American populations (OR = 1.00, 95% CI: 0.77–1.28). Maternal smoking was also associated with elevated ASD risk in studies with the lowest quartile of sample sizes (OR = 1.61, 95% CI: 1.12–2.33). Subgroup analyses by study design, study quality, and adjustment for confounders showed no significant differences in pooled ORs across strata. Assessment of maternal smoking at a prenatal visit (OR = 1.10, 95% CI: 1.03–1.17) or after birth (OR = 1.82, 95% CI: 1.37–2.42) showed significant associations with ASD in offspring, whereas assessment at birth (OR = 0.75, 95% CI: 0.65–0.85) had an apparent protective effect (Table 1). For the sensitivity analysis, the pooled ORs varied moderately, ranging from 1.08 (95% CI: 0.92–1.29) when Gao *et al*.^32^ was excluded to 1.17 (95% CI: 0.98–1.47) when Bilder *et al*.^25^ was excluded. Case deletion diagnostics identified Talbott *et al*.^22^ as a potential outlier. The main meta-analysis without Talbott *et al*. resulted in a similar pooled OR (OR = 1.12, 95% CI: 0.94–1.33) as the original analysis (OR = 1.16; 95% CI: 0.97–1.40).

**Table 1:** Summary of results from subgroup analyses.

Variable	No. of Studies	I^2^%	OR (95% CI)
**All Studies**	22	94.05%	1.14 (0.96–1.36)
**Location**			
Europe	9	0.02%	1.14 (1.08–1.19)
North America	12	91.69%	1.00 (0.77–1.28)
Asia	1		3.02 (1.93–4.73)
**Design**			
Case control	15	95.91%	1.22 (0.93–1.60)
Cohort	7	85.04%	1.04 (0.77–1.39)
**Adjusted Analysis**			
Yes	11	96.20%	1.09 (0.83–1.45)
No	11	91.15%	1.24 (0.96–1.60)
**Quality** ^**a**^			
High	4	92.90%	1.19 (0.65–2.15)
Medium	10	84.15%	1.00 (0.88–1.14)
Low	8	91.19%	1.32 (0.86–2.02)
**Sample Size** ^**b**^			
1Q	6	62.87%	1.61 (1.12–2.33)
2Q	5	94.17%	1.24 (0.88–1.73)
3Q	5	81.20%	1.04 (0.74–1.46)
4Q	6	83.63%	0.37 (0.67–1.22)
**Maternal Smoking Assessment**			
Prenatal	7	35.97%	1.10 (1.03–1.17)
At birth	4	53.26%	0.75 (0.65–0.85)
After birth	9	63.77%	1.82 (1.37–2.42)
**ASD Diagnosis**			
Direct evaluation	4	43.61%	1.29 (0.89–1.87)
Medical/Developmental record	16	96.55%	1.09 (0.88–1.37)
Parental report	2	0.00%	1.70 (1.23–2.35)

^a^Averaged NOS scores of 8–9 (high), 6–7 (medium), and 4–5.5 (low).

^b^Sample sizes of 208–870 (1Q), 871–10,309 (2Q), 10,310–55,993 (3Q), and 55,994–637,304 (4Q).

**Figure 4:**
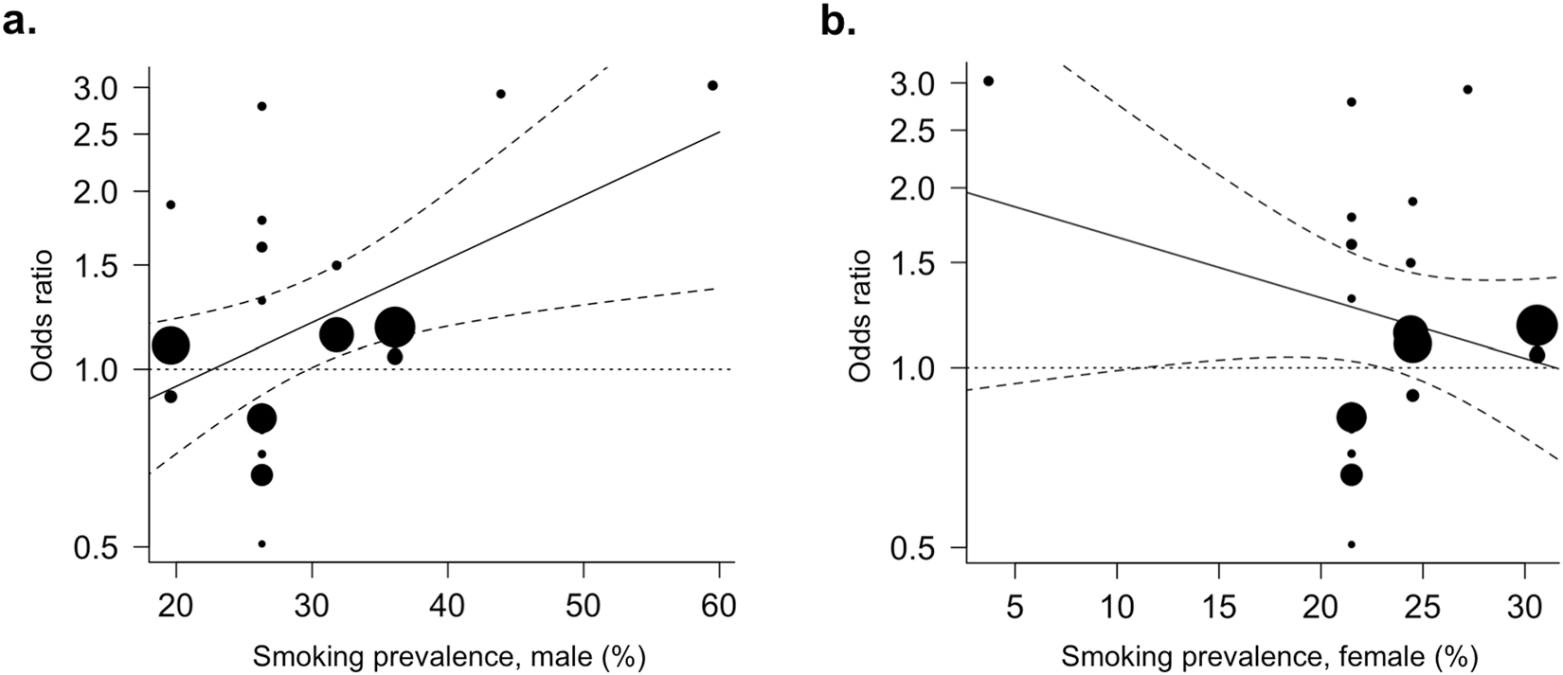
Meta-regression analysis using population smoking metrics as moderators. The OR of ASD with maternal smoking during pregnancy as moderated by (**a**) smoking prevalence in adult males (%) (z = 2.55, *p* = 0.011), and (**b**) smoking prevalence in adult females (%) (z = −1.13, *p* = 0.26) in the country of each study population. The diameters of the circles are proportional to study population size.

Interestingly, subgroup analysis using meta-regression with a continuous moderator found that smoking prevalence in males in the country of each study had a significant influence on ASD risk after maternal smoking (z = 2.33, p < 0.05). The results also showed that the OR increased with increasing smoking prevalance percentage. Therefore, we used the prediction function to obtain the average log OR values for the smoking prevalence percentages while holding the year constant. Smoking prevalence in males by country showed a significant correlation with ASD risk (z = 2.55, *p* = 0.011) (Fig. 4a). In contrast, smoking prevalence in females by country did not have a statistically significant correlation, (z = −1.13, *p* = 0.26) (Fig. 4b).

### Correlations between population smoking metrics and measures of secondhand smoke exposure

**Figure 5:**
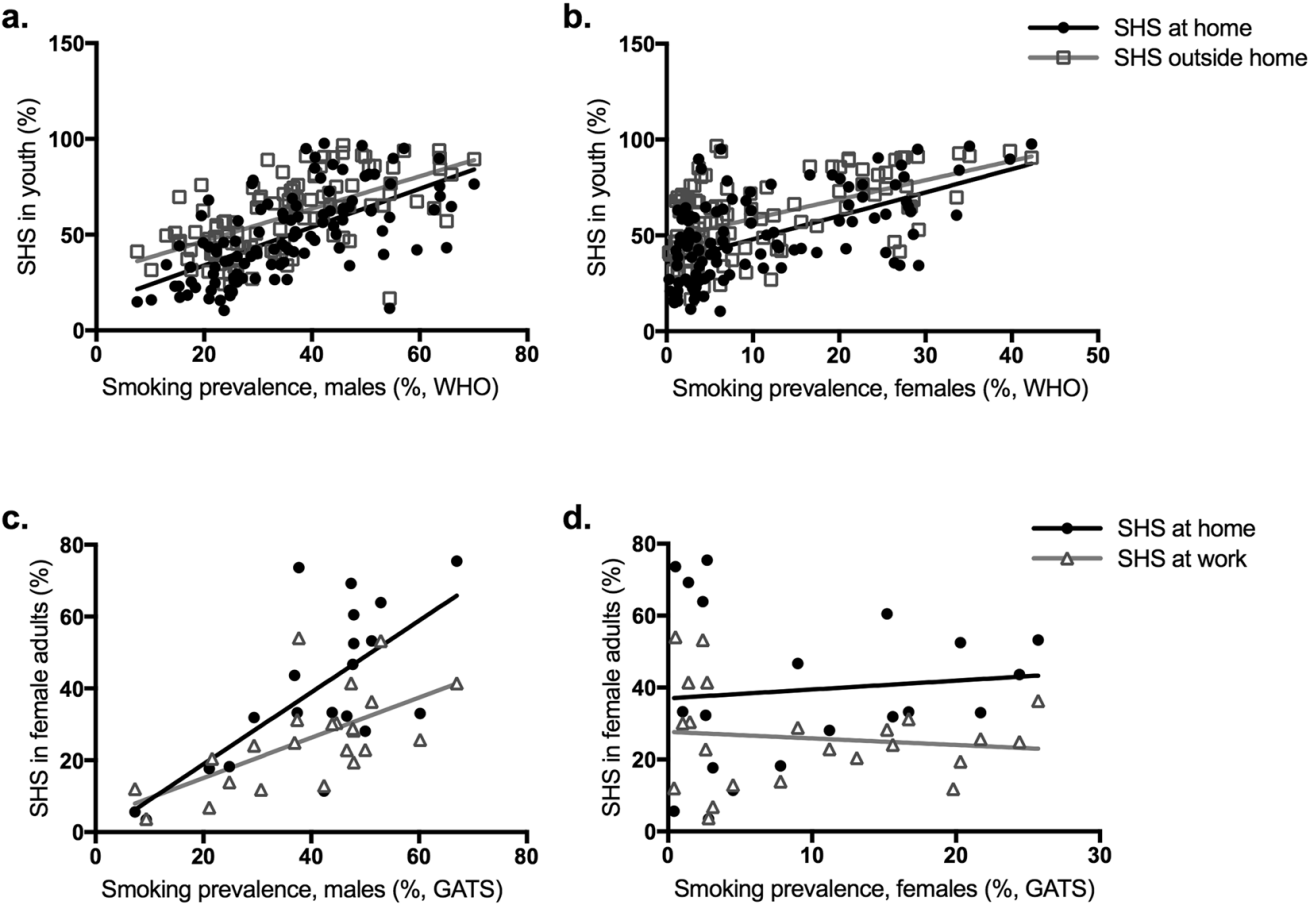
Correlation analyses of population smoking metrics and reports of secondhand smoke exposure. (**a**) Male and (**b**) female smoking prevalence percentages compared to secondhand smoke exposure reported by youths 13–15 years old for exposures at home and outside the home, all extracted from WHO’s 2008 report on the global tobacco epidemic. (**c**) Male and (**d**) female smoking prevalence percentages compared to secondhand smoke exposure reported by female adults for exposures at home and at work, according to the Global Adult Tobacco Survey accessed through CDC databases. SHS = secondhand smoke.

Reports of secondhand smoke exposure in either youth^48^ or in female adults^49^ were only available for three (U.S., China, Poland) of the eight countries represented in our study, precluding direct use of secondhand smoke measures in our meta-analyses. Therefore, we performed linear regression analyses to test the relationship between the population smoking metrics used in our analysis and the secondhand smoke exposure data we extracted from global survey reports. These analyses showed significant positive correlations between smoking prevalence in adult males with youth reports of smoke exposure in the home (*r*(108) = 0.63, *p* < 0.0001) and outside the home (*r*(106) = 0.61, *p* < 0.001) (Fig. 5a). Smoking prevalence in adult females also had significant correlations with youth reports of smoke exposure in the home (*r*(108) = 0.58, *p* < 0.0001) and outside the home (*r*(106) = 0.55, *p* < 0.0001) (Fig. 5b). Similar results were found when comparing male smoking prevalence to adult female reports of secondhand smoke exposure at home (*r*(18) = 0.70, *p* = 0.0006) and at work (*r*(21) = 0.64, *p* = 0.0011) (Fig. 5c). There was no significant association between female smoking prevalence and adult female reports of secondhand smoke exposure at home (*r*(18) = −0.011, *p* = 0.96) or at work (*r*(21) = −0.17, *p* = 0.44) (Fig. 5d).

## Discussion

The present study used a comprehensive search strategy and explored the relationship between maternal smoking during pregnancy and ASD in offspring. Three previously published meta-analyses have shown no significant association between maternal smoking and ASD in offspring, analyzing 5 or 15 individual studies^29–31^. Our main analysis, which included 22 studies and is the first to include an observational study in an Asian population, also showed no significant association between maternal smoking during pregnancy and ASD (Fig. 3). To determine the source of the substantial heterogeneity in our main analysis, we performed subgroup analyses using meta-regression with various modalities. We produced novel findings by comparing results by study location and by using population-level measures of tobacco consumption. In subgroup analyses, a significant positive association between maternal smoking and ASD in the offspring was observed in studies conducted in China and Europe, but not those in North America. Correlating with these relative rankings, the smoking population of adult males in China and the European countries represented in this analysis, except Sweden, are higher than the adult male smoking populations in the U.S. and Canada (Table S2). Our meta-regression analysis with adult male smoking prevalence in each country as a continuous moderator also indicated a significant correlation with ASD risk (Fig. 4a). Furthermore, adult male smoking prevalence was strongly correlated with rates of secondhand smoke exposure reported by youth and adult women (Fig. 5). These results suggest that maternal tobacco exposure may indeed increase ASD risk in offspring, but that exclusively assessing active maternal smoking underestimates total exposure, which includes secondhand smoke, before or after birth. Measures of smoking in the overall population may provide this missing measure of secondhand smoke exposure, thereby modulating the relationship between maternal smoking and ASD in offspring to produce significant correlations.

Two important limitations of using the smoking prevalence data should be noted. First, the 2008 smoking prevalence data were used for all subjects included in the meta-analysis, who were born between 1980–2012. The 2008 report was chosen because it was the earliest report to comprehensively and systematically measure smoking prevalence in member countries. However, our meta-analytic approach and the wide range of birth years represented makes it impossible to precisely match population smoking indices to the subjects’ gestation. Indeed, we cannot exclude the possibility that male smoking may contribute to exposure after birth, not during gestation. Future studies could more accurately track this information by collecting information regarding secondhand smoke exposure directly from the mothers in observational studies. Secondly, the trend towards a negative correlation between OR of ASD risk and smoking prevalence in women, although not statistically significant, seems incongruent with the idea that this metric captures an important measure of secondhand smoke exposure. However, we found that female smoking prevalence, compared to male smoking prevalence, was less strongly correlated with secondhand smoke exposure (Fig. 5). It is also possible that these results may be skewed by the extremely low smoking prevalence in females in China, where only 3.7% of women reported current smoking, compared to 21.5–30.6% of women in other countries^48^. Such low smoking rates in women have also been observed in other Asian countries such as South Korea, where 7.1% of women smoked according to in-person self-report surveys, but 18.2% of women smoked according to urine cotinine content^50^. In comparison, 47.8% of men smoked according to self-reports, and 55.1% of men smoked according to urine cotinine content. There may be a similarly disproportionate degree of underreporting in Chinese women, especially around pregnancy^51^, thereby skewing the data towards a negative trend.

An alternate explanation is that the significant correlations between OR and male smoking prevalence indicate a primary effect of paternal smoking on ASD in offspring, rather than through secondhand smoke exposure in the mother. There is evidence that smoking induces significant changes of DNA methylation in sperm^52, 53^. DNA methylation is associated with changes in the gene expression profile. *In utero* nicotine exposure in mice has been shown to induce such changes in gene expression through epigenetic modification^11^. Therefore, it would not be surprising if both maternal and paternal smoking contribute to changes in gene expression underlying brain development. However, three studies included in our meta-analysis examined secondhand smoke exposure during pregnancy, and their findings suggest that smoking increases ASD risk in offspring primarily through *in utero* exposure^19, 32, 41^. Furthermore, two other studies conducted in Chinese populations reported positive associations between passive maternal smoke exposure and ASD^54, 55^. The passive smoke exposure in these studies was not exclusive to paternal smoking and included, for example, exposure from smoking colleagues; active maternal smoking data was not reported. Together, these results suggest that paternal smoking may be an important factor for its impact on maternal smoke exposure during pregnancy, rather than for direct effects on the father’s role in reproduction.

Importantly, population-level smoking is one of many possible risk factors for ASD. Results of our subgroup and moderator analyses indicated that population-level tobacco consumption, study location, and study sample size could only account for 14% of the total heterogeneity found in this analysis. As noted in the introduction, there are several adverse outcomes, such as low birth weight, linked to both maternal smoking and to ASD, that not only suggest maternal smoking is a plausible environmental cause of ASD, but also pose potential confounds for studying the direct association between ASD and maternal smoking. Several observational studies included in our meta-analysis adjusted for potential confounding factors, either by matching controls to cases on characteristics of interest (e.g., birth year and gender)^25, 32^, or by adjusting their statistical analysis for potential confounds (e.g., parental age, parental education, parental occupational class, family income, maternal origin of birth, and Apgar score)^18, 41^. Our study examined adjustment for confounding factors as a potential moderator and, similar to others^30, 31^, found no differences in our subgroup analyses. However, contributions of these factors cannot be entirely ruled out.

One significant result from our subgroup analyses was that maternal smoking was associated with elevated ASD risk in studies with the lowest quartile of sample sizes (OR = 1.61, 95% CI: 1.12–2.33) (Table 1). Interestingly, all six studies in the lowest quartile of sample sizes assessed maternal smoking exposure after birth; subgroup analyses showed that this method of maternal smoking assessment, used in 9/22 studies, was also associated with an increased risk of ASD (OR = 1.82, 95% CI: 1.37–2.42) (Table 1). Additionally, all 4/22 studies that diagnosed ASD by direct evaluation are represented in the lowest quartile of samples sizes. The significance of this observation is unclear; it may be that studies directly evaluating ASD had small sample sizes necessitated by limited resources, and that the significant effect of sample size in our subgroup analysis is due to sampling bias. Overall, there is still a large portion of heterogeneity in our analyses that is not accounted for.

There are also general limitations to consider regarding ascertainment of exposure and case definition. First, half of the studies (11/22) relied on retrospective reporting, which many believe may be more vulnerable to faulty recall and bias than prospective reporting, especially if reported after an ASD diagnosis^30^. In both this and a previous meta-analysis^30^, subgroup analyses showed that assessing maternal smoking at birth had an apparent protective effect, while assessment after birth produced the highest OR for ASD risk (Table 1). Interestingly, whereas Rosen *et al*.^30^ found that prenatal assessment resulted in no significant relationship between maternal smoking and ASD, our study found a small but significant effect in the prenatally assessed subgroup of studies (OR = 1.10, 95% CI: 1.03–1.17). Empirically, studies have shown that retrospective reporting is overall accurate and reliable^56, 57^. One study found that retrospective recall of any smoking in pregnancy, an average of 14.5 years later, had 95.6% and 80.3% sensitivity when compared to positive urine cotinine tests during pregnancy and prospective reporting at the first antenatal visit, respectively, and 93.2% and 92.9% specificity, respectively, when including only women who recalled any smoking after learning of pregnancy^58^. Importantly, the study also found that there were no differences in child behavior problems, specifically conduct disorder symptoms and antisocial behaviors, between mothers’ whose retrospective recall of smoking status was or was not congruent with prospective reporting or cotinine levels. Therefore, the number of studies relying on retrospective reporting in our meta-analysis is likely not a significant issue, and the results of the subgroup analyses in prenatally and postnatally assessed mothers may accurately reflect a positive association. Second, while retrospective timing of reporting may not be uniquely susceptible to bias, self-reporting of maternal smoking may be particularly underreported if women are aware of its adverse health consequences and/or perceive social disapproval^51^.

More importantly, self-reporting of active smoking alone likely misrepresents total nicotine exposure. In China, 57% of women reported exposure to secondhand smoke^59^. In New York City, 46.8% (102/218) of pregnant women who were nonsmokers were found to have serum cotinine levels indicating secondhand smoke exposure^60^. The results of our moderator analyses indicate a significant relationship between the risk of ASD in children whose mothers’ smoked during pregnancy and male population smoking prevalence, which in turn was significantly correlated with reports of secondhand smoke exposure in youth and female adults. Together, these data may suggest that reports of active smoking alone neglects to account for nicotine exposure from secondhand smoking, which when accounted for may provide new insight into relationships between maternal exposures and subsequent child outcomes.

Another potential limitation is our broad definition of maternal smoking as any smoking during pregnancy, which may have skewed our main analysis towards a null association if *in utero* nicotine exposure must meet some threshold to increase ASD risk. Studies of attention-deficit/hyperactivity disorder risk after exposure to maternal smoking in pregnancy suggest there may be such a dose effect^61^; if also true for ASD, it would align with our interpretation that population smoking metrics capture secondhand smoke exposure, which increases total *in utero* nicotine dose and therefore significantly predicts ASD risk. In the observational studies included in our meta-analysis, only Haglund *et al*.^20^ reported smoking dosage^20^.

Limitations are also inherent in determining case or control status. Our subgroup analyses showed no differences in ORs when ASD status was determined by direct evaluation or by medical or developmental records; parental reporting of ASD status, in the only two studies using this method, resulted in a positive association (OR = 1.70, 95% CI: 1.23–2.35) (Table 1). Diagnoses were variably based on DSM-III, DSM-IV, DSM-IV-TR, or DSM-V criteria. Our definition for positive ASD status was also broad and included all subtypes, such as Asperger’s and Persistent Developmental Disorder, if reported separately. Additionally, in one study, we used a group with intellectual disability (ID) but without an ASD diagnosis as the control group, and ASD with or without ID as the case group^24^. It is unclear whether the various ASD subtypes have different etiologies, either in general or specifically in relation to maternal smoking^62^. Future studies that more rigorously and consistently distinguish between subtypes may yield more information.

In summary, our study included 22 observational studies and is the first to include one conducted in an Asian population^32^. While our main meta-analysis is consistent with previous meta-analyses in finding no significant association between maternal smoking and ASD in offspring, our subgroup and moderator analyses with population smoking metrics suggest that a more nuanced interpretation of this null association is warranted. In future studies, more consistent and systematic examination of non-maternal sources of *in utero* or developmental nicotine exposure, such as smoking in the fathers, household, or general population, may yield important results.

## Methods

### Data Sources and Searches

We followed PRIMSA-P guidelines^63^ for meta-analytic studies. Literature searches were conducted on PubMed, EMBASE, Web of Science, and Cochrane Library to identify peer-reviewed studies published through May 2016 with the following search term: [(smoke ∪ smoking ∪ cigarette ∪ tobacco ∪ risk factors) ∩ (autism spectrum disorder ∪ autism) ∩ (pregnancy ∪ prenatal ∪ perinatal ∪ Maternal ∪ Paternal ∪ Parental)]. No language restrictions were applied. Additionally, studies used in previously published meta-analyses were evaluated for inclusion. Country-specific data on population smoking metrics were extracted from World Health Organization (WHO) reports of adult smoking prevalence for the year 2008, separated by sex^48^. Smoking was defined as any current smoking at the time of data collection, and age-standardized estimates of smoking prevalence were extracted to allow comparison between countries. Data on secondhand smoke exposure were extracted from the Global Youth Tobacco Survey as reported in the same 2008 WHO report from which adult smoking prevalence data were extracted. Exposure to smoke was defined as “during the last seven days prior to the survey, people smoked at least once in the presence of the interviewee,” and was reported by youths 13–15 years old for exposure inside the home and outside the home^48^. The Center for Disease Control and Prevention’s (CDC) Global Tobacco Surveillance System (GTSS) data portal was used to access additional data from the Global Adult Tobacco Survey (GATS), from which data on adult reports of secondhand smoke exposure and prevalence of any current smoking in males and females were extracted by country^49^. Since our study focuses on maternal smoking, we extracted data on female adults’ reports of exposure to secondhand smoking at home (any smoking in home) and at work (in the past 30 days).

### Study Selection

Full texts of potentially relevant reports were examined for the following inclusion criteria: (i) a cohort or case-control design and (ii) data reported in such a way that the authors could extract or calculate the number of ASD cases with mothers who did and did not smoke during pregnancy, and control subjects with mothers who did and did not smoke during pregnancy. Two authors (YJ, AML) independently confirmed fulfillment of inclusion criteria.

### Data Extraction and Quality Assessment

Information was extracted from each study as a contingency table using a binary maternal smoking variable and a binary ASD variable. Any maternal smoke exposure during pregnancy was considered positive for maternal smoking, and any ASD in offspring, regardless of subtype, was considered positive for ASD. Two authors (YJ, AML) independently extracted data and resolved any disagreements by discussion. Additionally, the following data were extracted: last name of the first author, publication year, study design, study location, maternal smoking assessment method, ASD status assessment method, and unadjusted and adjusted odds ratios (ORs) with 95% confidence interval (CI), or alternate statistic if reported, and whether any adjustments for confounders were made in either participant selection or statistical analysis.

Two authors (YJ, AML) used the Newcastle-Ottawa Scale (NOS) to assess methodological quality^64^. A maximum of nine points was assigned for eight items in three categories, Selection, Comparability, and Exposure. First, each author independently determined preliminary NOS scores for each study, which were discussed to arrive at a consensus definition of the eight items to be scored. Subsequently, the authors independently re-scored each study according to the agreed-upon criteria, and averaged their scores.

### Data Analysis

Unadjusted ORs were calculated from the contingency tables abstracted from each study and combined to produce a measure of the association between maternal smoking and ASD risk. Cochrane Q statistic (significance level at *p* < 0.10) and the *I* ^2^ statistic were used to assess the heterogeneity of studies^65, 66^. A random-effects model was used to calculate the pooled OR in the context of a general linear model. Smoking prevalence in adult males or in adult females was used as a continuous moderator in the mixed-effects model to examine to what extent these moderators influence the strength of the relationship between maternal smoking and autism. A sensitivity analysis was performed to assess whether any individual study significantly affected pooled estimates by omitting one study in each turn using meta-regression fitting. Outliers or influencers in the studies were diagnosed by examining studentized deleted residuals (rstudent), difference of predicted average effect (DIFFITS), and total changes in parameter estimate (Cook’s distance) in meta-regression fitting. Additionally, subgroup analyses were performed to examine study design (case-control or cohort), location (by continent), sample size (by quartile), quality (NOS score high, medium, or low), adjustment for confounders (yes/no), timing of maternal smoking assessment (prenatal visit, at birth, after birth), and method of ascertaining ASD diagnosis (direct evaluation, medical or developmental record, parental report). Begg’s rank correlation and Egger’s regression tests were used to detect publication bias^46^. Metafor^67^ R package was used for all meta-analyses. GraphPad Prism version 7.0 was used to calculate Pearson’s correlation coefficient, *r*, to test for correlation between two authors’ independent NOS ratings, and to test for correlations between population smoking metrics and measures of secondhand smoke exposure. The correlation between female smoking prevalence and female adult smoke exposure based on GATS data was computed as a nonparametric Spearman correlation due to the non-Gaussian distribution of data. All statistical tests had a significance level of *p* *≤* 0.05, unless otherwise specified.

### Statistics

To determine the effect of moderators, a mixed-effects model was used, in which the unknown true effect, *y*_*i*_, is given by *y*_*i*_ = *β*_*0*_ + *β*_1_ *x*_*i*1_ + … + *β*_*p*_ *x*_*i*p_ + *u*_*i*_ + *e*_*i*_, where *x*_*i*p_ denotes the value of the *p*th moderator for the *i*th study, and *β*_*p*_ denotes the change in the average true effect for a one unit increase in *x*_*i*p_. The Metafor package is advantageous because it allows such mixed-effects models to be fitted with continuous or categorical *x* moderator variables, compared to previous packages that only allow categorical moderator variables to be fitted. Additionally, *u*_*i*_ ~ *N*(0, τ^2^) where τ^2^ denotes the residual heterogeneity not accounted for by the moderators included in the model, and *e*_*i*_ ~ *N*(0, *v*_*i*_) where *v*_*i*_ denotes sampling variances. Some assumptions underlying the meta-analysis conducted by Metafor in general include (1) treating sampling variances as if they were known constants, (2) treating τ^2^ as a constant after it has been estimated and (3) assuming effect sizes are normally distributed^67^. Further explanation of this model can be found in the Metafor package manual^68^ and clinical vignette^67^.

## Electronic supplementary material

Supplementary Tables
