# Supplementary material for: Maternal smoking and autism spectrum disorder: meta-analysis with population smoking metrics as moderators
Source: Sci Rep. 2017 Jun 28;7:4315. doi: 10.1038/s41598-017-04413-1 (PMC5489536; doi:10.1038/s41598-017-04413-1)
Supplement: Supplementary file 1 — Supplementary Tables [file 41598_2017_4413_MOESM1_ESM.docx]

**Table S1**. Description of the 22 studies included in the meta-analysis.

| **Authors (Year)** | **Continent** | **Country** | **MS Assessment** | **ASD**  **Diagnosis** | **Avg NOS Score** | **Sample Size** |
| --- | --- | --- | --- | --- | --- | --- |
| Williams *et al.* (2003) | North America | US | after birth | direct evaluation | 5 | 208 |
| Larsson *et al.* (2005) | Europe | Denmark | prenatal visit | medical record | 9 | 6048 |
| Maimburg and Væth (2006) | Europe | Denmark | prenatal visit | medical record | 7 | 4129 |
| Bilder *et al.* (2009) | North America | US | at birth | medical record | 8 | 13320 |
| Larsson *et al.* (2009) | Europe | Sweden | after birth | parental report | 5 | 4641 |
| Burstyn *et al.* (2010) | North America | Canada | at birth | medical record | 6 | 216342 |
| Dodds *et al.* (2011) | North America | Canada | prenatal visit | medical record | 7 | 125989 |
| Haglund and Källén (2011) | Europe | Sweden | prenatal visit | medical record | 5 | 60331 |
| Hvidtjørn *et al.* (2011) | Europe | Denmark | prenatal visit | medical record | 7 | 586306 |
| Volk *et al.* (2011) | North America | US | after birth | direct evaluation | 5.5 | 563 |
| Kalkbrenner *et al.* (2012) | North America | US | at birth | medical/develop-mental record | 6 | 637304 |
| Lee *et al.* (2012) | Europe | Sweden | prenatal visit | medical record | 7 | 42980 |
| Mrozek-Budzyn *et al.* (2013) | Europe | Poland | after birth | medical record | 5 | 288 |
| Roberts *et al.* (2013) | North America | US | after birth | parental report | 5 | 22351 |
| Tran *et al.* (2013) | Europe | Finland | prenatal visit | medical record | 9 | 20205 |
| Visser *et al.* (2013) | Europe | The Nether-lands | after birth | direct evaluation | 6 | 502 |
| Schmidt *et al.* (2014) | North America | US | after birth | direct evaluation | 6 | 851 |
| Gao *et al.* (2015) | Asia | China | after birth | medical record | 5 | 926 |
| Kalkbrenner *et al.* (2015) | North America | US | not specified | medical/develop-mental record | 6 | 13071 |
| Schieve *et al.* (2015) | North America | US | at birth | medical/develop-mental record | 4 | 7547 |
| Talbott *et al.* (2015) | North America | US | after birth | medical record | 8 | 430 |
| Xiang *et al.* (2015) | North America | US | not specified | medical record | 6 | 64924 |

**Table S2.** Description of the 22 studies included in the meta-analysis by the variables used in subgroup analyses and the meta-regression moderator variables.

| **Authors (Year)** | **Continent** | **Country** | **Design** | **Adj. analysis** | **Quality^a^** | **Sample Size^b^** | **SP in Men (%)** | **SP in Women (%)** |
| --- | --- | --- | --- | --- | --- | --- | --- | --- |
| Williams *et al.* (2003) | North America | US | CC | yes | L | 1Q | 26.3 | 21.5 |
| Larsson *et al.* (2005) | Europe | Denmark | CC | yes | H | 2Q | 36.1 | 30.6 |
| Maimburg and Væth (2006) | Europe | Denmark | CC | yes | M | 2Q | 36.1 | 30.6 |
| Bilder *et al.* (2009) | North America | US | CC | yes | H | 3Q | 26.3 | 21.5 |
| Larsson *et al.* (2009) | Europe | Sweden | COH | no | L | 2Q | 19.6 | 24.5 |
| Burstyn *et al.* (2010) | North America | Canada | COH | yes | M | 4Q | NA | NA |
| Dodds *et al.* (2011) | North America | Canada | COH | no | M | 4Q | NA | NA |
| Haglund and Källén (2011) | Europe | Sweden | CC | yes | L | 4Q | 19.6 | 24.5 |
| Hvidtjørn *et al.* (2011) | Europe | Denmark | COH | no | M | 4Q | 36.1 | 30.6 |
| Volk *et al.* (2011) | North America | US | CC | no | L | 1Q | 26.3 | 21.5 |
| Kalkbrenner *et al.* (2012) | North America | US | CC | yes | M | 4Q | 26.3 | 21.5 |
| Lee *et al.* (2012) | Europe | Sweden | CC | yes | M | 3Q | 19.6 | 24.5 |
| Mrozek-Budzyn *et al.* (2013) | Europe | Poland | CC | yes | L | 1Q | 43.9 | 27.2 |
| Roberts *et al.* (2013) | North America | US | COH | no | L | 3Q | 26.3 | 21.5 |
| Tran *et al.* (2013) | Europe | Finland | CC | yes | H | 3Q | 31.8 | 24.4 |
| Visser *et al.* (2013) | Europe | The Nether-lands | CC | no | M | 1Q | 38.3 | 30.3 |
| Schmidt *et al.* (2014) | North America | US | CC | no | M | 1Q | 26.3 | 21.5 |
| Gao *et al.* (2015) | Asia | China | CC | yes | L | 2Q | 59.5 | 3.7 |
| Kalkbrenner *et al.* (2015) | North America | US | COH | no | M | 3Q | 26.3 | 21.5 |
| Schieve *et al.* (2015) | North America | US | CC | no | L | 2Q | 26.3 | 21.5 |
| Talbott *et al.* (2015) | North America | US | CC | No | H | 1Q | 26.3 | 21.5 |
| Xiang *et al.* (2015) | North America | US | COH | No | M | 4Q | 26.3 | 21.5 |

CC = case-control study; COH = cohort study; SP = smoking prevalence

1. Low (L), medium (M) and high (H) based on averaged NOS scores of 4-5.5, 6-7, and 8-9, respectively.
2. Sample sizes of 208-870 (1Q), 871-10,309 (2Q), 10,310-55,993 (3Q), and 55,994-637,304 participants (4Q).
